# Results of interferon-based treatments in Alaska Native and American Indian population with chronic hepatitis C

**DOI:** 10.3402/ijch.v75.30696

**Published:** 2016-03-29

**Authors:** Stephen E. Livingston, Lisa J. Townshend-Bulson, Dana J. T. Bruden, Chriss E. Homan, James E. Gove, Julia N. Plotnik, Brenna C. Simons, Philip R. Spradling, Brian J. McMahon

**Affiliations:** 1 Liver Disease and Hepatitis Program, Alaska Native Tribal Health Consortium, Anchorage, AK, USA; 2 Arctic Investigations Program, Division of Preparedness and Emerging Infections, National Center for Emerging and Zoonotic Infectious Diseases, Centers for Disease Control and Prevention, Anchorage, AK, USA; 3 Division of Viral Hepatitis, National Center for HIV/AIDS, Viral Hepatitis STD, and TB Prevention, Centers for Disease Control and Prevention, Atlanta, GA, USA

**Keywords:** pegylated interferon, discontinuation, indigenous population, longitudinal study, sustained virologic response

## Abstract

**Background:**

There have been few reports of hepatitis C virus (HCV) treatment results with interferon-based regimens in indigenous populations.

**Objective:**

To determine interferon-based treatment outcome among Alaska Native and American Indian (AN/AI) population.

**Design:**

In an outcomes study of 1,379 AN/AI persons with chronic HCV infection from 1995 through 2013, we examined treatment results of 189 persons treated with standard interferon, interferon plus ribavirin, pegylated interferon plus ribavirin and triple therapy with a protease inhibitor. For individuals treated with pegylated interferon and ribavirin, the effect of patient characteristics on response was also examined.

**Results:**

Sustained virologic response (SVR) with standard interferon was 16.7% (3/18) and with standard interferon and ribavirin was 29.7% (11/37). Of 119 persons treated with pegylated interferon and ribavirin, 61 achieved SVR (51.3%), including 10 of 46 with genotype 1 (21.7%), 38 of 51 with genotype 2 (74.5%) and 13 of 22 with genotype 3 (59.1%). By multivariate analysis, SVR in the pegylated interferon group was associated with female sex (p=0.002), estimated duration of infection (p=0.034) and HCV genotype (p<0.0001). There was a high discontinuation rate due to side effects in those treated with pegylated interferon and ribavirin for genotype 1 (52.2%). Seven of 15 genotype 1 patients treated with pegylated interferon, ribavirin and telaprevir or boceprevir achieved SVR (46.7%).

**Conclusions:**

We had success with pegylated interferon-based treatment of AN/AI people with genotypes 2 and 3. However, there were low SVR and high discontinuation rates for those with genotype 1.

The approval and use of direct-acting antiviral agents (DAAs) for the treatment of chronic hepatitis C virus (HCV) infection has dramatically improved efficacy and safety as well as decreased treatment duration. However, pegylated interferon may still be used, although it is no longer the standard of care for treatment.

The first treatment licensed in the United States and other countries for hepatitis C was standard interferon, which required subcutaneous injections thrice weekly. In the 1990s, this was combined with ribavirin and resulted in an increase in efficacy. From 2001 to 2011, the standard of care for hepatitis C treatment was pegylated interferon and ribavirin, with duration of 48 weeks for genotype 1 and 24 weeks for genotypes 2 and 3. Many studies on pegylated interferon and ribavirin reported overall sustained virologic response (SVR) rates of 40–45% in genotype 1 and 70–80% in genotypes 2–3, defining SVR as undetectable serum HCV RNA 24 weeks after the end of treatment (1,2). The majority of patients in these studies were Caucasian. Among non-whites, higher SVR rates in genotype 1 were reported in Asian Americans (3–5) compared with North American Hispanics and African Americans (3,5–8). This difference is partially explained by the distribution of the IL28B gene in different ethnic populations, which is strongly related to response (9). The advent of triple therapy for genotype 1 infection in 2011 with the addition of first-generation protease inhibitors increased SVR rates up to 70–75% in clinical trials but resulted in a worsened side effect profile and greater discontinuation (10,11).

Few reports of treatment results in indigenous populations are published, including Alaska Native and American Indian (AN/AI) people. We retrospectively examined our treatment results with interferon-based regimens in a cohort of AN/AI people living in Alaska.

## Methods

### Alaska Hepatitis C Cohort (AK-HepC)

In 2013, Alaska reported approximately 143,000 AN/AI (labor.alaska.gov). The Alaska Native Tribal Health Consortium, Liver Disease and Hepatitis Program (ANTHC LDHP) maintains a clinical registry of AN/AI people who are infected with HCV. There are 3,118 AN/AI persons identified as anti-HCV antibody seropositive in the ANTHC LDHP Hepatitis C Registry; of those, 2,573 are confirmed HCV positive by either recombinant immunoblot assay or HCV RNA. The majority of these people live in urban areas. Due to potential serious adverse events of interferon-based therapy and the non-availability of lab testing, few people were treated outside of these urban areas. As previously described, routes of hepatitis C transmission identified by participants included intravenous drug use (60.1%) and blood transfusion (14.1%) (12), with similar risk factors and viral genotype distribution to the National Health and Nutrition Examination Survey (13).

The ANTHC LDHP has enrolled 1,379 AN/AI persons into a long-term outcomes cohort study of chronic HCV infection that began in 1995; the AK-HepC study outcomes through 2013 were examined. All AN/AI people positive for anti-HCV and HCV RNA in the registry were invited to join AK-HepC study. Genotype distributions in this cohort were: 64% for genotype 1, 20% for genotype 2, 15% for genotype 3 and <1% for genotype 4.

Treatment decisions were made based on treatment eligibility factors. These included but were not limited to patient preference, no report of drug and alcohol use in the 6 months, amount of liver fibrosis, absence of poorly controlled psychiatric conditions or concurrent medical conditions, and absence of decompensated cirrhosis (14,15). Prior to 2001, individuals were treated with either standard interferon alone or standard interferon and ribavirin for 24–48 weeks. Starting in 2001, all individuals received pegylated interferon alfa-2a or alfa-2b plus ribavirin according to American Association for the Study of Liver Diseases (AASLD) guidelines (16). Individuals with genotype 1 were treated with pegylated interferon, ribavirin and a protease inhibitor after the approval of boceprevir and telaprevir in mid-2011, also according to AASLD guidelines (17).

All individuals involved in this study provided informed written consent. This study was approved by the Alaska Area and Centers for Disease Control and Prevention Institutional Review Boards and the Board of Directors of the Alaska Native Tribal Health Consortium and Southcentral Foundation.

### Laboratory testing

Testing for HCV RNA was performed until 2007 as previously described (18). Since 2007 testing for HCV RNA has been performed either at Quest Diagnostics (Seattle, WA) or at the Alaska Native Medical Center laboratory using the COBAS^®^ AmpliPrep/COBAS^®^ TaqMan^®^ HCV Test Kit (Roche Molecular Systems, Inc., USA), with an initial lower limit of quantification of 43 IU/mL and current lower limit of 15 IU/mL. HCV genotyping was performed as described previously (19), and since 2008 by HCV genotype LIPA, RT-PCR and reverse hybridization of the 5’ UTR and core region of the HCV genome (Quest Diagnostics, Seattle, WA). Alanine aminotransferase (ALT) testing was performed at the ANMC laboratory (Abbott Laboratories, Abbot Park, IL, USA) or in regional health care facilities. An ALT level of ≤40 U/L was defined as normal.

### Statistical analysis

We examined the association of SVR with patient characteristics for individuals treated with pegylated interferon and ribavirin. We defined SVR as undetectable HCV RNA 24 weeks after the end of treatment. We also examined the association of SVR with the achievement of rapid virologic response (RVR), which we defined as undetectable HCV RNA at 4 weeks of treatment. We used the likelihood ratio chi-square test to compare SVR in dichotomous variables. We used the Cochran–Mantel–Haenszel test when controlling for HCV genotype. We used logistic regression to test for associations between continuous covariates and SVR. For a multivariate variable model in an intention to treat analysis, we considered covariates with a univariable p<0.25. The model was built using purposeful forward selection and the Wald chi-square statistic. All p-values were two-sided and a value less than 0.05 was considered statistically significant. All analyses were performed using SAS version 9.3 (SAS Institute, Cary, NC).

We did not perform statistical analysis for those treated with standard interferon-containing regimens (interferon alone or interferon with ribavirin) or with triple therapy including a protease inhibitor because of small sample size in these groups.

## Results

### Treatment with standard interferon

Between 1992 and 1998, 18 persons were treated with standard interferon for 48 weeks and 3 achieved SVR (16.7%, Table 1). This included 1 of 10 with genotype 1 and 1 of 6 with genotype 3. Genotype was unknown on an additional person who achieved SVR and results were not available on one person with genotype 2 who was lost to follow-up. Three of nine females and none of 8 males with known results achieved SVR. Mean age at the time of treatment was 40.2 years. Demographic data were otherwise limited on this group.

**Table 1:** Hepatitis C treatment sustained virologic response by regimen and genotype in a cohort of Alaska Native and American Indian people

Treatment regimen	Number treated	SVR (%)^a^	Genotype 1 SVR	Genotype 2 SVR	Genotype 3 SVR
Interferon	18^b^	3 (16.7)	1/10 (10%)	0/1 (0%)	1/6 (16.7%)
Interferon/ribavirin	37	11 (29.7)	2/12 (16.7%)	3/9 (33.3%)	6/16 (37.5%)
Pegylated interferon/ribavirin^c^	119^d^	61 (51.3)	10/46 (21.7%)	38/51 (74.5%)	13/22 (59.1%)
Pegylated interferon/ribavirin/telaprevir or boceprevir^e^	15	7 (46.7)	7/15 (46.7%)	–	–

a SVR=Sustained virologic response, defined as undetectable HCV RNA 24 weeks after the end of treatment and expressed as intention to treat results.

b Includes 1 person with genotype 2 whose treatment results were unknown. Genotype on 1 person who achieved SVR was unknown.

c Eight persons were treated twice with pegylated interferon/ribavirin and results from first treatment course are reported. Three achieved SVR with the second treatment course.

d Includes 2 persons who achieved an end-of-treatment response but were lost to follow-up.

e Five of twelve persons treated with telaprevir (41.7%) and 2 of 3 treated with boceprevir (66.7%) achieved SVR.

### Treatment with standard interferon and ribavirin

Between 1997 and 2002, 37 persons were treated with standard interferon and ribavirin. Those with genotype 1 were treated for 48 weeks; those with genotypes 2 and 3 were treated for 24 weeks. Eleven achieved SVR (29.7%, Table 1). SVR results by genotype were as follows: 2 of 12 with genotype 1 (16.7%); 3 of 9 with genotype 2 (33.3%) and 6 of 16 with genotype 3 (37.5%). Two persons with genotype 1 had been previously treated with standard interferon. One of those achieved SVR. Three persons with genotype 3 had been previously treated with standard interferon and none of these achieved SVR with standard interferon and ribavirin. None of the 9 persons with genotype 1a achieved SVR versus 2 of 3 with genotype 1b. Six of 17 females achieved SVR (35.3%) and 5 of 20 males achieved SVR (25%). Mean age at the time of treatment was 43 years for both males and females.

### Treatment with pegylated interferon and ribavirin

#### Baseline characteristics

A total of 139 treatment regimens with pegylated interferon and ribavirin were started on 119 patients with genotypes 1, 2 and 3, of whom 114 (95.8%) were treatment naïve. Genotype 1 treatment length was 48 weeks; genotypes 2 and 3, 24 weeks. Genotype distribution was as follows: genotype 1, 46 (38.7%); genotype 2, 51 (42.9%); and genotype 3, 22 (18.5%). Nine persons were treated twice and 3 persons were treated 3 times. Of those re-treated, 8 were treated twice with pegylated interferon and ribavirin. Treatment results and patient characteristics were analysed on the first pegylated interferon and ribavirin treatment course for these 8 persons. The others were previously treated with standard interferon or standard interferon and ribavirin. Two persons were HIV positive and were included in the results. Two persons included in the results were hepatitis B surface antigen positive. Gender distribution was nearly equal, with 59 males (49.6%) and 60 females (50.4%). Mean age at treatment initiation was 44.7 years (44.2 males, 45.1 females). Mean body mass index (BMI) was 29.4 (29.0 females, 29.7 males). Mean ALT at the start of treatment was 105.7 (range 15–629) and was available for 117 patients. Mean HCV RNA at the start of treatment was 4,096,347 IU/mL and was available for 107 patients. Liver biopsies were performed on 78 patients prior to treatment and 22 (28.2%) had bridging fibrosis or cirrhosis (Table 2). Duration of infection could be estimated on 110 patients, with the following results by 10-year intervals: <10, 29 patients; 10–20, 31 patients; 20–30, 27 patients; and >30, 23 patients.

**Table 2:** Baseline characteristics of Alaska Native and American Indian people treated with pegylated interferon and ribavirin

Characteristic	Overall (%)	Genotype 1 (%)	Genotype 2 (%)	Genotype 3 (%)
Number treated	119	46 (38.7)	51 (42.9)	22 (18.5)
Male gender	59 (49.6)	19 (41.3)	29 (56.9)	11 (50)
Female gender	60 (50.4)	27 (58.7)	22 (43.1)	11 (50)
Mean age, years^a^	44.7	43.8	45.4	44.6
Mean BMI^a^,^b^	29.4	30.0	28.4	30.4
Mean ALT^a^,^c^	105.7	96.7	121.4	87.5
Mean HCV RNA^a^,^d^	4,096,347	2,516,909	6,821,071	1,341,314
Liver biopsy	78	39	26	13
Advanced fibrosis^e^	22 (28.2)	10 (25.6)	7 (26.9)	5 (38.5)

a Mean of characteristic at start of treatment.

b BMI=body mass index.

c ALT=alanine aminotransferase; available for 117 of 119 patients.

d HCV RNA, IU/mL, available for 107 of 119 patients.

e Advanced fibrosis on liver biopsy, defined as Ishak 3–6.

#### Overall treatment results

Of 119 persons, 61 achieved SVR (51.3%, Table 1). This includes 2 persons who had end-of-treatment undetectable HCV RNA but were then lost to follow-up. Thirty-nine persons discontinued treatment due to side effects (32.8%) and 12 failed treatment (10.1%). Excluding the 2 persons who lost to follow-up, 8 of the 117 with results available relapsed (6.8%). Of the 8 persons treated twice with pegylated interferon and ribavirin, 3 achieved SVR during the second treatment. Of the 2 HIV-positive persons, one, with genotype 1a, achieved SVR and the second, with genotype 2b, failed treatment.

Treatment was discontinued by the provider in 4 persons with retinal cotton wool exudates, including 2 with blurred vision and 1 with diabetes mellitus. Treatment was discontinued in 2 persons due to hyperthyroidism, including one also with atrial fibrillation. Anaemia was common, with 23 persons whose haemoglobin decreased to <10 gm/dL, including 6 with haemoglobin <8.5 gm/dL.

Ribavirin dose reductions were made in 31 persons, primarily for anaemia, 2 for nausea and vomiting and 1 for an elevated creatinine. Eighteen of these also had pegylated interferon dose reduction, due primarily to neutropenia. Of these 31 persons, 17 achieved SVR. Another 13 persons had dose reductions of pegylated interferon only and 8 of these achieved SVR. Seven people were treated with erythropoietin for anaemia.

#### Genotype 1 results

Of 46 persons (21.7%) with genotype 1, 10 achieved SVR when treated with pegylated interferon and ribavirin (Table 3). This included 7 of 34 with genotype 1a (20.6%) and 3 of 8 with genotype 1b (37.5%, p=0.36). Four persons had either genotype 1a/1b or could not be subtyped and none of those achieved SVR. The majority of those who did not achieve SVR discontinued treatment due to side effects (24 of 46, 52.2%), whereas 9 were treatment failures (19.5%) and 3 relapsed (6.5%). If those who discontinued due to side effects are omitted, 10 of 22 achieved SVR. Multiple side effects were reported by those who discontinued treatment, including flu-like symptoms, fatigue, nausea and vomiting, depression, anxiety and pain. These changes and the blurred vision resolved after treatment discontinuation (45.5%).

**Table 3:** Results of pegylated interferon–ribavirin treatment of Alaska Native and American Indian people

Genotype		Number	SVR^a^ (%)	Failure^b^ (%)	Relapse^c^ (%)	Discontinued^d^ (%)
1a		34	7 (20.6)	5 (14.7)	3 (8.8)	19 (55.9)
1b		8	3 (37.5)	2 (25)	0 (0)	3 (37.5)
Other^e^		4	0 (0)	2 (50)	0 (0)	2 (50)
	Total	46	10 (21.7)	9 (19.5)	3 (6.5)	24 (52.2)
2		51^f^	38 (74.5)	3 (5.9)	3 (6.1)	9^g^ (17.6)
3		22^f^	13 (59.1)	1 (4.5)	2 (9.1)	6 (27.3)

a SVR=Sustained Virologic Response, defined as undetectable HCV RNA 24 weeks after the end of treatment.

b Failure=<2-log drop of HCV RNA at week 12 or detectable HCV RNA at end-of-treatment course.

c Relapse=detectable HCV RNA within 6 months after undetectable at the end of treatment.

d Discontinued=treatment discontinued due to side effects.

e Other=genotype 1a/1b (2) or no subtype identified (2).

f Includes 2 persons with genotype 2 who achieved end-of-treatment response and then were lost to follow-up.

g Includes 2 who achieved SVR despite discontinuation.

Testing for RVR was performed in 25 patients, and 6 achieved RVR (40%). Of these, 2 achieved SVR (33%) and 4 discontinued treatment due to side effects. Of the 19 who did not achieve RVR, 2 achieved SVR (10.5%, p=0.23 compared to those who achieved RVR), 8 discontinued due to side effects, 7 failed treatment and 2 relapsed.

#### Genotype 2 results

Of 51 persons started on pegylated interferon and ribavirin for genotype 2, 38 achieved SVR (74.5%, Table 3). This group includes 2 persons who completed treatment but were lost to follow-up after achieving an end-of-treatment response. Nine persons discontinued treatment due to side effects (17.6%) and 2 of these achieved SVR; 3 were treatment failures (6.1%) and 3 relapsed after achieving an end-of-treatment response (6.1%).

Of 36 persons tested, 29 achieved RVR (80.6%). Of those who achieved RVR, 26 achieved SVR (89.7%), whereas only 2 of 7 persons who did not have an RVR achieved SVR (28.6%, p=0.003).

#### Genotype 3 results

Of 22 persons started on pegylated interferon and ribavirin for genotype 3, 13 achieved SVR (59.1%, Table 3). Six persons discontinued treatment due to side effects (27.3%); 2 relapsed (9.5%) and 1 failed treatment (4.5%).

Of 14 persons tested, 8 achieved RVR (57.1%); these 8 also achieved SVR. All 6 who did not achieve RVR did not achieve SVR also (p<0.001 compared to those who achieved RVR).

#### Effect of patient characteristics on treatment response

Univariate analysis was performed comparing persons achieving SVR versus those not achieving SVR. Only HCV genotype (p<0.01), baseline haemoglobin level (p=0.03), and estimated duration of infection by 10-year intervals (p=0.02) were associated with treatment response (Table 4). IL28B genotype was available in only 20 persons with genotype 1. Six persons were IL28B genotype CC and 2 of these achieved SVR, whereas none of the 14 persons with genotypes CT or TT achieved SVR (p=0.08). IL-28b testing was not conducted in this cohort until 2011. Therefore, the numbers were too small for further analysis. By multivariate analysis, SVR was associated with female sex (p=0.03, OR 3.1, CI 1.2–8.2); estimated duration of HCV infection (p=0.03, OR for a 10-year increase 1.5, CI 1.0–2.2), and HCV genotype (p<0.0001) (Table 5). Odds ratio for SVR comparing genotype 2 versus 1 was 14.0 (CI 4.6–42.4) and for genotype 3 versus 1 was 7.1 (CI 2.0–25.1).

**Table 4:** Factors related to hepatitis C treatment sustained virologic response^a^ in Alaska Native and American Indian people treated with pegylated interferon and ribavirin

Factor	Level	Number	SVR (n)	P
Sex	Female	60	55% (33)	0.17
	Male	59	43% (25)	
Age	<40 years	31	45% (14)	0.39
	40–49 years	40	43% (17)	
	50+ years	48	56% (27)	
HCV genotype	1	46	22% (10)	<0.01
	2	51	69% (35)	
	3	22	59% (13)	
Body mass index	<25	29	38% (11)	0.40
	25–29	40	53% (21)	
	30+	50	52% (26)	
Diabetes–pre-diabetes	Yes	41	54% (22)	0.29
	No	65	43% (28)	
Estimated duration of HCV infection	<10 years	29	38% (11)	0.02
	10–20 years	31	35% (11)	
	20–30 years	27	59% (16)	
	30+ years	23	65% (15)	
Baseline HCV RNA (IU/mL)	<500,000	33	55% (18)	0.14
	≥500,000–<3,000,000	36	44% (16)	
	≥3,000,000	38	37% (14)	
Advanced fibrosis (Ishak≥3)	Yes	22	45% (10)	0.43
	No	56	36% (20)	
Baseline ALT level^b^	<40	24	58% (14)	0.46
	40–<80	40	45% (18)	
	80–<120	21	48% (10)	
	120+	33	45% (15)	
Baseline Hgb level^c^	<14	30	60% (18)	0.03
	≥14	39	33% (13)	
Baseline ANC level^d^	<4	30	50% (15)	0.52
	≥4	22	41% (9)	
Baseline platelet count^e^	<200	36	42% (15)	0.35
	200–<300	45	51% (23)	
	≥300	36	53% (19)	
	≥1.5	28	32% (9)	
TSH	<1.5	51	45% (23)	0.26
	>1.5	28	32% (9)	

a Sustained virologic response was defined as undetectable HCV RNA 24 weeks after the end of treatment.

b ALT=alanine aminotransferase, expressed as units/litre.

c Hgb=haemoglobin, expressed in grams/decilitre.

d ANC=absolute neutrophil count, 10^9^ per litre.

e Platelet count, 10^3^ per microliter.

**Table 5:** Factors significantly related by multivariate analysis to hepatitis C treatment sustained virologic response in Alaska Native and American Indian people treated with pegylated interferon and ribavirin

Factor	Multivariate P-value	Comparison	Odds ratio of SVR (confidence interval)
Genotype	<0.0001	2 vs. 1	14.0 (4.6, 42.4)
		3 vs. 1	7.1 (2.0, 25.1)
Gender	0.03	Female vs. male	3.1 (1.2, 8.2)
Estimated duration of HCV infection	0.03	10-year increase	1.5 (1.0, 2.2)

a Sustained virologic response (SVR) was defined as undetectable HCV RNA 24 weeks after the end of treatment.

### Treatment with pegylated interferon, ribavirin and first-generation protease inhibitors

A total of 15 persons with genotype 1 received triple therapy with pegylated interferon, ribavirin and telaprevir or boceprevir. Of these, 6 had been previously treated with pegylated interferon and ribavirin. Those receiving triple therapy included 8 females and 7 males, with mean age 48.7 years, mean BMI 34.1 and mean HCV RNA at start 3,148,150 IU/mL, including 13 with HCV RNA >400,000 IU/mL. Twelve of 14 persons with liver biopsy results had bridging fibrosis or cirrhosis. IL28B genotype was tested on 14 persons and showed 4 with CC, 8 CT and 2 TT. Eleven persons had genotype 1a; 2 had genotype 1b and 2 could not be subtyped.

Seven of 15 persons achieved SVR (46.7%, Table 1). Five of 12 persons (41.7%) treated with telaprevir achieved SVR, compared with 2 of 3 (66.7%) treated with boceprevir. Six persons achieved RVR, and 5 of those also achieved SVR. Three persons were eligible for response-guided therapy and all 3 achieved SVR with a 24-week course. Of the 8 persons treated with triple therapy who did not achieve SVR, 4 failed treatment and 4 discontinued due to side effects.

Notable side effects included 6 patients on telaprevir who developed a rash during weeks 1–3, none of whom required discontinuation due to the rash. In addition, 9 patients on telaprevir developed haemoglobin <10 during treatment, including 3 with haemoglobin <8.5. Two were treated with erythropoietin and 1 of these 2 also received a blood transfusion. Four persons required a decrease in pegylated interferon dosage during treatment.

Of those previously treated with pegylated interferon and ribavirin, one also was treated with consensus interferon and ribavirin and relapsed both times before achieving SVR with telaprevir triple therapy. One person achieved SVR with boceprevir triple therapy. Of the other 4 persons, 2 failed triple therapy and 2 discontinued due to side effects.

## Discussion

Overall hepatitis C treatment results for AN/AI people with standard interferon-containing regimens were generally poor, with SVR rates of 16.7% for standard interferon alone and 29.7% for standard interferon plus ribavirin. This was similar to SVR rates reported in a 1999 literature review (20), for a 48-week treatment course with these early regimens.

We found SVR rates with pegylated interferon and ribavirin of only 21.3% in genotype 1 patients. This is much lower than results from clinical trials, (1,2,21) and represents the real-life experience of interferon-based treatment (22). These results are similar to those reported in African Americans, including the IDEAL trial, which reported a 22% SVR for African Americans compared to 44% for Caucasians (5). Two other trials found SVR rates in African Americans significantly lower than Caucasians (6,7). Dropout rates due to side effects for African Americans in these 2 trials were 19 and 22%, respectively, and those in several large trials were reported to be 10–15% (1,2), whereas a US Veterans Administration study reported that only 22% of over 10,000 patients completed a treatment course for hepatitis C (20). In our cohort, 48.9% (23 of 47 persons) with genotype 1 discontinued treatment due to side effects. This high dropout rate may partially explain the low SVR rates. If those who discontinued treatment due to side effects are omitted, our SVR rate was 41.7%. SVR rates in Asian Americans have been reported to be 56–65%, which is higher than for other ethnic groups, but only 35–38% in Hispanic Americans (3–5). We did not find a statistically significant influence of IL28B genotype on our HCV genotype 1 SVR rate, as we had IL28B data on only 20 of 46 participants. As in a “real-life” setting, the Alaska Hepatitis C cohort is comprised of a genetically heterogenous population, thus we are unable to determine whether treatment outcomes are due to specific genetic factors in this study population.

We found only 3 other reports in the literature of hepatitis C treatment results in indigenous populations. One of these was a prospective trial of pegylated interferon and ribavirin treatment in 46 Canadian Aboriginal persons and 55 Caucasians with genotype 1 (23). Investigators found a statistically significant lower SVR rate in Aboriginal people (35% vs. 55%, p=0.047). Premature treatment termination, baseline normal ALT values and fatty liver on ultrasound were associated with outcome on multivariate analysis. Another Canadian study retrospectively examined pegylated interferon and ribavirin treatment results in 44 persons who identified themselves as Aboriginal Canadians in a large nationwide study (24). Overall SVR rates were 47.7% in Aboriginal and 46.5% in non-Aboriginal patients. Results were also similar between the groups by genotype. Finally, in a brief report, 22 Australian indigenous persons (18 were genotype 1) were started on pegylated interferon and ribavirin and 4 of 8 with results available achieved SVR (25). Results were not reported by genotype.

Observed SVR rates in genotype 2 and 3 patients were slightly lower than those reported in some studies. SVR rates for our cohort were 74.5% for genotype 2 and 59.1% for genotype 3, whereas other studies have reported overall SVR rates for genotypes 2 and 3 combined to be about 80% (1,2,20,26). We had relatively high dropout rates due to side effects for these genotypes, 17.6% for genotype 2 and 27.3% for genotype 3.

By multivariate analysis we found that female sex, estimated duration of HCV infection by 10-year increase and genotype were independently associated with SVR in individuals treated with pegylated interferon and ribavirin. The association of female sex with SVR has not often been reported (1,27) although one small study found such an association, primarily in women under age 40 (28). If estimated duration of infection were considered a surrogate marker for fibrosis, then individuals with a longer duration of infection might be expected to have more advanced fibrosis and a lower SVR rate. We cannot explain why the SVR rate increased with estimated duration of infection and the literature does not appear to address this. As previously published results have shown dramatic differences in treatment eligibility among this cohort, perhaps these pre-existing factors influenced the resulting treatment outcome (14).

Our treatment of genotype 1 patients with triple therapy was primarily limited to individuals who previously failed pegylated interferon and ribavirin treatment and/or who had advanced fibrosis. Although we did not perform statistical evaluation of these results due to small numbers, our treatment experience was similar to others in terms of patient side effects (29).

Limitations of this study relate to its retrospective nature and limited demographic data in the early years of treatment prior to the advent of pegylated interferon use. In addition, we did not have a comparison group in our study and thus we could only compare our results to those from other studies.

In conclusion, we have had success with interferon-based treatment of AN/AI population with chronic hepatitis C with genotypes 2 and 3. However, we have had high discontinuation and low SVR rates in genotype 1. We are now actively treating individuals with newer DAA regimens that are better tolerated, have a shorter duration of treatment and have significantly higher SVR rates (30). The arrival of interferon-free DAA treatment is improving access to hepatitis C treatment for Alaska Native and American Indian people living in Alaska.
